# Atrial fibrillation after electrical cardioversion in elderly patients: a role for arterial stiffness? Results from a preliminary study

**DOI:** 10.1007/s40520-016-0620-8

**Published:** 2016-08-27

**Authors:** Stefano Fumagalli, Ilaria Giannini, Simone Pupo, Francesca Agostini, Serena Boni, Anna T. Roberts, Debbie Gabbai, Claudia Di Serio, Luciano Gabbani, Francesca Tarantini, Niccolò Marchionni

**Affiliations:** 1 Geriatric Intensive Care Unit, Experimental and Clinical Medicine Department, University of Florence and AOU Careggi, Florence, Italy; 2 School of Nursing, University of Florence, Florence, Italy

**Keywords:** Arterial stiffness, Atrial fibrillation, CAVI, CHA_2_DS_2_-VASc, Elderly, Electrical cardioversion

## Abstract

**Background and aims:**

Atrial fibrillation (AF) is the most frequent arrhythmia of the elderly, and electrical cardioversion (ECV) is a common procedure, although incidence of recurrences remains high. We evaluated the possible association between arterial stiffness (AS) and the persistence or recurrence of AF in elderly patients after ECV.

**Methods:**

We enrolled all subjects undergoing ECV over a 9-month period. AS was evaluated with the cardio-ankle vascular index (CAVI). Patients were then visited at follow-up (on average at 6 months).

**Results:**

Thirty-one patients (age 78 ± 7 years; men 67.7 %; CHA_2_DS_2_-VASc 4.1 ± 1.6; AF length >2 months 51.6 %; CAVI 9.9 ± 1.6) underwent ECV. At follow-up, sinus rhythm was recorded in 16 (51.6 %) patients. At multivariate analysis, the presence of AF was directly associated with CHA_2_DS_2_-VASc score and CAVI. Amiodarone therapy reduced the risk of relapsed AF.

**Conclusions:**

In elderly AF patients treated with ECV, AS at baseline seems to predict AF at follow-up.

## Introduction

Atrial fibrillation (AF) is the most common sustained rhythm disorder of the elderly [1]. Arterial stiffness (AS) increases with age and predicts coronary heart disease, stroke and mortality [2]. In a previous pilot study on AF patients, we found that AS could represent an important factor associated with left atrium (LA) remodeling [3]. In the present study, we evaluated whether AS has a role in sinus rhythm maintenance in elderly subjects undergoing elective electrical cardioversion (ECV) of persistent AF. We also examined concentrations of interleukin-6 (IL-6), which are associated with AS in healthy adults [4] and early AF recurrences [5].

## Methods

### Patients and procedures

We enrolled all patients admitted to our day-hospital for ECV between March and December 2015. At baseline, all patients underwent assessment of physical and neuro-cognitive function with the short physical performance battery (SPPB) and the mini-mental state examination (MMSE). Depressive symptoms were evaluated with the geriatric depression scale (GDS). Venous IL-6 concentrations were determined in 27 out of 31 (87.1 %) patients using commercially available ELISA kits (R&D Systems, Inc.; Minneapolis, MN, USA). ECV was performed using a biphasic defibrillator, after a 4-week period of effective oral anticoagulation [6].

The cardio-ankle vascular index (CAVI) [3, 7], a measure of AS, independent from instantaneous systolic and pulse pressure, was evaluated immediately before discharge using VaSera VS-1500N (Fukuda Denshi, Japan). During the continuous recording of EKG and heart sounds, right and left upper and lower extremity arterial pressure was obtained with the oscillometric method. Pulse wave velocity (PWV) was calculated dividing the distance from the aortic valve to the ankle by the sum of two time intervals (1. aortic valve closing sound–notch of the brachial pulse wave; 2. rise of the brachial pulse wave–rise of the ankle pulse wave) [7]. CAVI was then computed using the following equation:

$${\text{CAVI}} = a \times \left[ {\left( {2\rho /\Delta P} \right) \times { \ln }\left( {P_{\text{s}} /P_{\text{d}} } \right) \times {\text{PWV}}^{2} } \right] + b$$

where *P*_s_/*P*_d_ are systolic/diastolic pressures, Δ*P* is “*P*_s_–*P*_d_”, *ρ* is blood density, and *a*/*b* are constants [7].

At follow-up, arterial blood pressure, EKG, SPPB, MMSE, GDS and main clinical events were recorded in all cases.

### Statistical analysis

IBM SPSS for Windows (version 23) was used for statistical analysis. Continuous and categorical variables are expressed as mean ± SD and numbers with percentages, respectively. Linear regression analysis described the relation between continuous variables. Student’s *t* test and analysis of variance—or the related nonparametric tests—were used to compare continuous variables between groups. The association between categorical variables was evaluated with Chi-square test. Logistic regression analysis models identified clinical predictors of sinus rhythm at follow-up. A two-tailed *p* value <0.05 was considered statistically significant.

## Results

### Study population

Thirty-eight patients underwent elective ECV. We excluded 7 subjects (18.4 % of the whole population) because of ECV of an arrhythmia relapse (*N* = 3), age <55 years, severe sinus node dysfunction, logistic reasons and refusal to participate (*N* = 1 each). Characteristics of the 31 patients included in the study are reported in Table 1.

**Table 1:** Main characteristics in all patients and by AF at follow-up

	All patients	AF at follow-up		*p*
		No	Yes	
Continuous variables				
Age (years)	78 ± 7	74 ± 8	81 ± 4	0.005
Height (cm)	170 ± 9	170 ± 8	171 ± 9	0.593
Weight (Kg)	74 ± 14	75 ± 13	73 ± 15	0.637
MMSE (score)	27.6 ± 3.4	28.4 ± 1.5	26.9 ± 4.4	0.243
GDS (score)	3.4 ± 1.8	3.4 ± 1.6	3.4 ± 2.3	0.980
SPPB (total score)	9.6 ± 2.2	9.8 ± 2.4	9.4 ± 2.0	0.424
CHA_2_DS_2_-VASc (score)	4.1 ± 1.6	3.3 ± 1.4	5.0 ± 1.4	0.003
HR (bpm)	74 ± 14	73 ± 15	74 ± 14	0.803
SAP (mmHg)	134 ± 21	127 ± 20	141 ± 20	0.036
DAP (mmHg)	77 ± 11	75 ± 10	80 ± 11	0.178
Left atrium diameter (mm)	52 ± 4	52 ± 4	52 ± 5	0.709
IVS thickness (mm)	10 ± 1	10 ± 1	10 ± 1	0.839
LVEDD (mm)	50 ± 7	51 ± 6	49 ± 7	0.308
LVEF (%)	61 ± 9	61 ± 9	61 ± 8	0.980
Categorical variables (*N*, %)				
Men	21 (67.7)	11 (68.8)	12 (66.7)	1.000
Living alone	6 (19.4)	2 (12.5)	4 (26.7)	0.394
Smokers (present/past)	16 (51.6)	8 (50.0)	8 (53.3)	1.000
Wine (> 1 glass/day)	19 (61.3)	10 (62.5)	9 (60.0)	1.000
CAD	9 (29.1)	4 (25.0)	5 (33.3)	0.704
CHF	15 (48.4)	7 (43.8)	8 (53.3)	0.724
Chronic renal failure	3 (9.7)	1 (6.3)	2 (13.3)	0.600
COPD	3 (9.7)	0 (0)	3 (20.0)	0.101
CVD	4 (12.9)	2 (12.5)	2 (13.3)	1.000
Diabetes	8 (25.8)	1 (6.3)	7 (46.7)	0.015
Dyslipidemia	14 (45.2)	5 (31.3)	9 (60.0)	0.156
Hypertension	27 (87.1)	12 (75.0)	15 (100)	0.101
PAD/aortic aneurism	5 (16.2)	1 (6.3)	4 (26.7)	0.172
Thyroid dysfunction	9 (29.0)	4 (25.0)	5 (33.3)	0.704
AF length >2 months	16 (51.6)	4 (25.0)	12 (80.0)	0.004
ACE-I/ARBs	27 (87.1)	14 (87.5)	13 (86.7)	1.000
Beta-blockers	22 (71.0)	13 (81.3)	9 (60.0)	0.252
Statins	15 (48.4)	7 (43.8)	8 (53.3)	0.724
Digitalis	13 (41.9)	7 (43.8)	6 (40.0)	1.000
Amiodarone	14 (45.2)	11 (68.8)	3 (20.0)	0.011
Class IC AADs	4 (12.9 %)	1 (6.3)	3 (20.0)	0.333
OAT—warfarin	21 (67.7)	10 (62.5)	11 (73.3)	0.704
OAT—NOACs	10 (32.3)	6 (37.5)	4 (26.7)	

*ARBs* Angiotensin receptor blockers, *CAD* coronary artery disease, *CHF* chronic heart failure, *Class IC AADs* class I C antiarrhythmic drugs, *COPD* chronic obstructive pulmonary disease, *CVD* cerebrovascular disease, *GDS* geriatric depression scale, *HR* heart rate, *IVS* interventricular septum, *LVEDD* left ventricular end-diastolic diameter, *LVEF* left ventricular ejection fraction, *MMSE* mini-mental state examination, *OAT* oral anticoagulant therapy, *NOACs* non-VKA oral anticoagulants, *PAD/Aortic Aneurism* peripheral artery disease/aortic aneurism, *SAP/DAP* systolic/diastolic arterial pressure, *SPPB* short physical performance battery, *Wine* wine consumption or equivalent for other alcoholic beverages

Mean age was 78 years (33rd–66th percentile: 76–80 years). On the whole, neuro-cognitive function was preserved and prevalence of disability low.

Hypertension emerged as the most important risk factor for AF. Arrhythmia length was >2 months in 16 subjects (51.6 %). CHA_2_DS_2_-VASc score and AS were high. Only 7 patients (22.6 %) showed CAVI values <9 [7].

Most patients received antagonists of the renin–angiotensin system and beta-blockers; amiodarone was the most frequently used antiarrhythmic drug (Table 1).

### AF predictors at follow-up

ECV was effective in 90.3 % of patients (*N* = 28/31). At the follow-up evaluation (mean length: 179 days, 33rd–66th percentile: 104–252 days), sinus rhythm was observed in 51.6 % of population (*N* = 16).

**Fig. 1:**
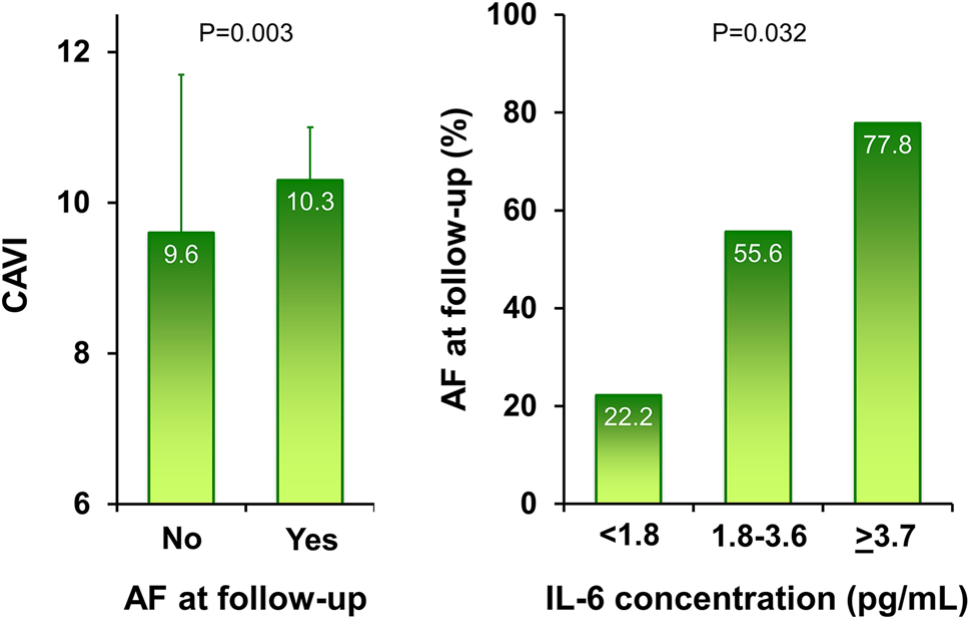
Arterial stiffness at baseline, measured with cardio-ankle vascular index (CAVI), by the presence of AF at follow-up (*left* *panel*) and proportion of patients with AF at follow-up by tertiles of interleukin-6 (IL-6) concentration (*right* *panel*). Because *right* and *left* CAVI values were not different (*p* = 0.872), we reported the left ones for their higher statistical association with the endpoint variable

At follow-up, patients presenting AF tended to be older, but no differences were observed according to gender, neuro-cognitive and functional profile, depressive symptoms and history of CHF. Prevalence of diabetes, CHA_2_DS_2_-VASc score, baseline systolic arterial pressure, AS and AF duration were all significantly higher in those with AF at follow-up (Table 1; Fig. 1). Regarding drugs, only amiodarone was associated with a higher persistence of sinus rhythm. Subjects with AF showed higher IL-6 concentrations (Fig. 1); a pattern between higher levels of the cytokine and age, diabetes, AS, CHA_2_DS_2_-VASc score, anti-gout therapy and a poor SPPB performance also emerged.

Multivariate logistic regression analysis demonstrated that the presence of AF at follow-up visit was directly correlated with the CHA_2_DS_2_-VASc score and AS, while amiodarone therapy maintained an independent protective association (Table 2).

**Table 2:** Multivariate predictors of the presence of AF at follow-up visit

	β ± e.s.	OR (95 % CI)	*p*
CHA_2_DS_2_-VASc (∆ point)	0.97 ± 0.49	2.65 (1.01–6.94)	0.048
CAVI (∆ unit)	0.84 ± 0.42	2.31 (1.01–5.25)	0.046
Amiodarone (yes vs. no)	−2.91 ± 1.30	0.05 (0.01–0.70)	0.025
Constant	−23.33 ± 10.54	/	0.027

Results of the multivariate logistic regression analysis (overall predictivity = 83.7 %)

Variables excluded from the model: baseline systolic arterial pressure (*p* = 0.101); AF length >2 months (*p* = 0.429)

Δ change in the dependent variable per unitary change in the independent variable, *CAVI* arterial stiffness assessed with the cardio-ankle vascular index, obtained through arterial pressure measures at the left arm and ankle, *OR* odds ratio

## Discussion

The results of the present study demonstrate that ECV is extremely effective also in elderly patients, with a success rate as high as 90 %. However, relapse is frequent; at follow-up, about 50 % of our population showed AF. The presence of the arrhythmia, due to new episodes and to previous ECV failures, was inversely associated with amiodarone therapy and directly related to cardio-embolic risk and to AS, as expressed by the CHA_2_DS_2_-VASc score and CAVI, respectively.

The efficacy of amiodarone in preventing AF recurrences has already been demonstrated. The “Canadian Trial of Atrial Fibrillation Investigators” showed that the incidence of arrhythmia relapses with amiodarone was lower than that observed with sotalol or propafenone, but the rate of adverse events was also higher [8]. More recently, a study, conducted in the Department of Veterans Affairs National Health Care System on subjects with a newly diagnosed AF, found that amiodarone use was not associated with increased mortality after adjustment for age, gender, the presence of CHF, renal function and use of beta-blockers and warfarin [9].

In patients undergoing electrical or pharmacological cardioversion, the CHA_2_DS_2_-VASc score emerged as a significant predictor of early recurrence of AF [10]. Similarly, in the “Leipzig Heart Center AF Ablation Registry,” the CHA_2_DS_2_-VASc score was associated with both early and late relapses of the arrhythmia [11]. Hypertension, diabetes and CHF, through their action on inflammation, oxidative stress and atrial fibrosis, could represent a link between the scores of cardio-embolic risk and AF recurrence [11]. Confirming this hypothesis, in our population, we found an inverse association between IL-6 concentration and the prevalence of sinus rhythm at the follow-up. The CHA_2_DS_2_-VASc score seems useful to identify AF patients with a higher overall risk of events. At this regard, in subjects with a mean age of 73.9 years, during a 2-year follow-up, scoring 9, when compared to 0, was associated with a three times higher risk to be admitted in hospital for cardiovascular causes [12].

Indeed, the most interesting finding of our preliminary experience was the direct association between AS and the presence of arrhythmia at follow-up. For each one-unit increase in CAVI, the risk of finding AF at the control visit was 2.31 times higher. Few previous reports, obtained with surrogate measures, enforced our results. After 4.9 years of follow-up, among patients with hypertension and left ventricular hypertrophy enrolled in the “Losartan intervention for endpoint (LIFE) reduction in hypertension study,” the incidence of a first episode of the arrhythmia was 4.0 %, with a robust, significant, relation with pulse pressure, a marker of AS [13]. Among the Framingham Heart Study participants aged ≥35 years, cumulative 20-year AF incidence rates were 5.6 and 23.3 % for pulse pressure values ≤40 and >61 mmHg, respectively. Hence, AS could play a role on AF-related mechanisms also in community-based cohorts [14].

Our results are the first to show that in an elderly population undergoing ECV of persistent AF, the presence of arrhythmia at follow-up, more often a recurrence, is directly related to arterial properties. A possible explanation could be represented by the association between CAVI and left atrium diameter, which is independent of left ventricular thickness [3]. Once again, low-grade inflammation could be the link between aortic stiffness and left atrium dilation [15].

The small number of patients is the major limitation of the study. IL-6 concentration was not measured in all subjects. This fact, coupled with the collinear increase in age, diabetes, AS and the CHA_2_DS_2_-VASc score, prevented us from adjusting our models by IL-6 values. However, this is a preliminary, pilot study. The results we obtained are biologically and clinically plausible and could represent the starting point for further, more detailed investigations.

 In conclusion, in an elderly AF population, the occurrence of arrhythmia at follow-up seems to be related not only to CHA_2_DS_2_-VASc score and the use of amiodarone, but also to AS. Hence, the modulation of vascular properties could represent a possible target to reduce AF burden and its complications in aged, at-risk subjects.
